# PP36 Real-world Effectiveness Of Umeclidinium And Umeclidinium/Vilanterol For Chronic Obstructive Pulmonary Disease: A Singapore Database Study

**DOI:** 10.1017/S0266462324002083

**Published:** 2025-01-07

**Authors:** Shannon Ng, Benjamin Ong, Kwong Ng

## Abstract

**Introduction:**

Long-acting muscarinic antagonist (LAMA) monotherapy and LAMA in combination with a long-acting beta2 agonist are preferred over inhaled corticosteroid/long-acting beta2-agonist (ICS/LABA) therapy for Global Initiative for Chronic Obstructive Lung Disease (COPD) Group B and C patients. This study assesses impact of the subsidy decision of umeclidinium (umec) and umeclidinium/vilanterol (umec/vil) on Singapore’s healthcare system.

**Methods:**

A retrospective cohort study was conducted using national health record databases. Maintenance-naive COPD patients, with no concurrent asthma, initiated on umec, umec/vil, or ICS/LABA from 2016 to 2020, were included. Patient demographics, comorbidities, and clinical characteristics were balanced using propensity score matching. Primary outcomes measured were the rate of severe or moderate COPD exacerbation and pneumonia hospitalization within one-year follow-up. Effect size was estimated using incidence rate ratio (IRR) with 95% confidence intervals (CIs) from Poisson regression. Markov model extrapolated the number of exacerbations and pneumonia hospitalizations avoided arising from the initiation of umec or umec/vil over ICS/LABA.

**Results:**

Patients initiated on umec (n=1,019) were less likely to experience severe (IRR 0.649; 95% CI: 0.438, 0.961) and moderate exacerbations (IRR 0.713; 95% CI: 0.569, 0.892) than ICS/LABA. Similarly, umec/vil-treated patients (n=1,206) had lower rates of severe (IRR 0.713; 95% CI: 0.517, 0.985) and moderate exacerbation (IRR 0.778; 95% CI: 0.642, 0.942). Both therapies were safer than ICS/LABA, with fewer pneumonia hospitalizations for umec (IRR 0.719; 95% CI: 0.532, 0.973) and umec/vil (IRR 0.781; 95% CI: 0.623, 0.980). Coupled with reduced drug cost from value-based pricing, subsidy potentially resulted in SGD53 million (USD39 million) cost savings over 10 years.

**Conclusions:**

As the largest real-world study conducted among COPD patients in Singapore, our findings contribute to the limited real-world evidence in the region. Compared to ICS/LABA, umec or umec/vil were associated with better COPD control and reduced rates of pneumonia hospitalization. This confirms the importance of appropriate prescribing of COPD therapies and validates the subsidy decision.

